# A Telerehabilitation Program Improves Postural Control in Multiple Sclerosis Patients: A Spanish Preliminary Study

**DOI:** 10.3390/ijerph10115697

**Published:** 2013-10-31

**Authors:** Rosa Ortiz-Gutiérrez, Roberto Cano-de-la-Cuerda, Fernando Galán-del-Río, Isabel María Alguacil-Diego, Domingo Palacios-Ceña, Juan Carlos Miangolarra-Page

**Affiliations:** Department of Physical Therapy, Occupational Therapy, Physical Medicine and Rehabilitation, Faculty of Health Sciences, Rey Juan Carlos University, Avda. Atenas s/n. Alcorcón 28922, Madrid, Spain; E-Mails: rosaortizg@hotmail.com (R.O.-G.); fernando.galandel@urjc.es (F.G.-R.); isabel.alguacil@urjc.es (I.M.A.-D.); domingo.palacios@urjc.es (D.P.-C.); juan.miangolarra@urjc.es (J.C.M.-P.)

**Keywords:** multiple sclerosis, postural control, telerehabilitation, video games

## Abstract

Postural control disorders are among the most frequent motor disorder symptoms associated with multiple sclerosis. This study aims to demonstrate the potential improvements in postural control among patients with multiple sclerosis who complete a telerehabilitation program that represents a feasible alternative to physical therapy for situations in which conventional treatment is not available. Fifty patients were recruited. Control group (*n =* 25) received physiotherapy treatment twice a week (40 min per session). Experimental group (*n =* 25) received monitored telerehabilitation treatment via videoconference using the Xbox 360^®^ and Kinect console. Experimental group attended 40 sessions, four sessions per week (20 min per session).The treatment schedule lasted 10 weeks for both groups. A computerized dynamic posturography (Sensory Organization Test) was used to evaluate all patients at baseline and at the end of the treatment protocol. Results showed an improvement over general balance in both groups. Visual preference and the contribution of vestibular information yielded significant differences in the experimental group. Our results demonstrated that a telerehabilitation program based on a virtual reality system allows one to optimize the sensory information processing and integration systems necessary to maintain the balance and postural control of people with multiple sclerosis. We suggest that our virtual reality program enables anticipatory PC and response mechanisms and might serve as a successful therapeutic alternative in situations in which conventional therapy is not readily available.

## 1. Introduction

Multiple sclerosis (MS) is a chronic inflammatory demyelinating disease of the central nervous system (CNS) of unknown aetiology and multifactorial origin [1]. MS is the most common chronic neurological disease in young adults in Europe and North America [1]. Balance and postural control (PC) disorders are among the most frequent motor disorder symptoms associated with MS, as these symptoms are present in 20% of patients with MS at onset and chronic in 80% of cases [2]. Most patients report that balance and gait difficulties are the leading causes of disability [1]. Balance and PC are closely related concepts that require the CNS integration of visual, vestibular, and somatosensory (proprioceptive and cutaneous) information as well as the proper activation of neuromuscular control mechanisms [3]. Thus, balance and PC disorders in patients with MS are associated with difficulty standing and performing functional activities, thereby significantly affecting quality of life [4]. Different authors have suggested that balance disorders are the leading cause of falls in people with MS, reporting an incidence rate between 52% and 63% from 2–6 months that increases with age and the progression of disability [4,5]. The risk of falling significantly decreases the mobility, social participation, and social interaction of patients with MS and has negative effects on their physical health and emotional states [5]. 

Neurorehabilitation programs are among the most popular therapies aimed at reducing the disabilities and social disadvantages that result from MS. The delivery of these services must be profitable, equitable, accessible, sustainable, and of high quality [6]. Many of the sequelae of neurological diseases are treated on an outpatient basis in hospitals and specialized centers. Importantly, these resources are limited and deficient in the clinical setting because of the time-constrained nature of rehabilitation. In addition, most patients with MS have difficulties related to mobility, geographical location, or both that prevent them from receiving treatment at a rehabilitation center. Furthermore, personnel and material resources are often needed to provide such treatment, which increases the cost of therapy and the difficulty of providing continuous treatment [7].

In response to this situation, interest has recently increased with regard to the development of eHealth projects. In the context of eHealth, telerehabilitation (TR) is the delivery of rehabilitation services via electronic systems using information and communication technologies (ICT) [8]. TR extends rehabilitative care beyond the hospital setting in an eco-friendly environment, helping to detect new limitations and evaluate the effectiveness of the intervention with regard to the activities of daily living (ADLs) at a sustainable cost [8]. Different platforms supporting online TR for patients with neurological disorders have allowed caregivers to address motor, cognitive, and care aspects remotely in diseases such as Parkinson’s disease, stroke, and spinal cord injury; however, their application remains rare in MS treatment [9]. These platforms enable patient monitoring, either asynchronously through forums, e-mail, wikis, or blogs or synchronously through chat servers, instant messaging, videoconferencing, collaborative browsing, or remote presentation [10]. Patient monitoring allows caregivers to follow up with the implementation and progress of therapy as well as modify or adjust a personalized program based on planned and accomplished objectives [10]. 

Among these technologies, virtual reality (VR), especially as a TR configuration system, has recently gained importance in the rehabilitation of patient with motor and cognitive neurological dysfunctions [11,12]. The major features of this multimedia technology are aimed at enabling interaction and sensory feedback in patients via a highly motivating multidimensional virtual environment in which the patient performs in virtual daily activities or tasks. Patients rank the intensity and difficulty of these tasks, thereby providing real-time information with regard to the achieved objectives [13]. 

Recently, studies related to the use of video game consoles have proliferated in the field of motor rehabilitation. This technology has showed favorable results in treating balance [14] and gait [15] disorders, the functionality and activity of the upper limb [16], and training patients with neurological to perform ADLs [17]. Interactive multimedia technologies offer certain advantages over traditional rehabilitation treatments because they provide the patient with an opportunity to practice ADLs that cannot occur in conventional rehabilitation environments either due to accessibility issues, geography, or treatment availability. These technologies can also provide motivational activities that facilitate therapeutic adherence and treatment compliance [18]. However, more research is needed on neurorehabilitation with regard to patients with MS. 

This study aims to demonstrate the potential improvements in balance and PC among patients with MS who complete a VR TR program that represents a feasible alternative to physical therapy for situations in which conventional treatment is not available.

## 2. Experimental Section

The Demyelinating Diseases Unit of the Neurology Department at San Carlos University Hospital, Madrid, recruited 50 patients with MS in according with the revised McDonald criteria. Recruitment was conducted using consecutive non-probability sampling and based on the inclusion criteria shown in Table 1.

**Table 1:** Inclusion and exclusion criteria.

Inclusion criteria	Exclusion criteria
(1) Age between 20 and 60 years (2) Confirmed diagnosis of MS for over 2 years based on McDonald criteria [19] (3) Medically stable during the 6 months prior to baseline (4) Impaired balance associated with demyelinated lesions in the cerebellum and its connections (5) EDSS score ranging from 3 to 5; (6) Hauser ambulatory index value higher than 4 (7) Absence of cognitive impairment according to the mini mental state examination test (8) No visual deficits (9) Internet connection at home. The level of experience with consoles and video games was not a criterion for the recruitment of patients.	(1) Diagnosed with another disease or pathological condition that affects balance (2) Had an attack in the month prior to baseline or during the intervention process (3) Received an intravenous or oral steroid cycle prior to beginning the evaluation protocol and within the 4-month duration of the project intervention

MS: Multiple Sclerosis; EDSS: Expanded Disability Status Scale.

According to criteria of availability and accessibility to different rehabilitation centers (MS Madrid Association and MS Madrid Foundation), and under an agreement with the hospital, patients who did not receive conventional physiotherapy treatment were included in the experimental group (EG) based on at least one the following criteria: (a) wait on the waiting list; (b) limited geographic accessibility; (c) unable to reconcile working hours and therapy schedule; or d) dependent on others to arrive at the treatment center. Each patient was evaluated at the hospital described above. Of the 50 participants enrolled, 23 met the inclusion criteria for the EG. The remaining participants (*n =* 27) were randomly distributed into two treatment groups using QuickCalcs from GraphPad Software^®^. Due to the equipment availability criterion, two participants were added to the EG; thus, 25 were included in the final sample.

The control group (CG; *n =* 25) received physiotherapy treatment twice a week (40 min per session) at the MS Madrid Association and Foundation. The treatment was based on low-loads strength exercises (10 min per session), propioception exercises on unstable surfaces and gait facilitation exercises (20 min per session), and, finally, muscle-tendon stretching (10 min per session). In all cases, fatigue self-perceived was taken into account with an analogue visual scale.

Participants in the EG (*n =* 25) received individual TR treatments using the Xbox 360^TM^ console with Microsoft^TM^ Kinect following a protocol specifically designed for this purpose. These sessions were monitored via videoconference. Kinect uses a set of infrared sensors to recognize the physical position and size of the patient. An RGB camera gathers facial recognition data, and a multi-array microphone detects voice and extracts ambient sound. The Kinect system enables users to create a digital skeleton (*i.e*., avatar) via its software. Participants use 3D motion-capture technology to control their avatar via hands-free bodily movement.

Currently, commercial software is not available for the Xbox 360TM for the application of rehabilitation therapy, specifically, for treating patients with neurological balance disorders. Thus, the current treatment program was developed using existing recreational and commercial gaming software designed for entertainment and leisure. Physiotherapists from the Department of Physical Therapy, Occupational Therapy, Rehabilitation, and Physical Medicine at King Juan Carlos University (Madrid, Spain) with experience in rehabilitating patients with MS and who were familiar with the use of video games designed the gaming protocol. An exercise protocol was initially developed from seven games that are compatible with the Xbox 360^TM^ and Kinect^TM^; three games were discarded due to their gaming difficulty and high degree of physical requirements. A second protocol was designed and tested in participants with MS with similar characteristics to the sample; an additional game was discarded because it caused excessive fatigue in participants. The final conclusive and reliable treatment gaming protocol consisted of three games (Kinect Sports^TM^, Kinect Joy Ride^TM^, and Kinect Adventures^TM^).

The activities included in the gaming protocol were based on the recommendations of Cattaneo *et al*. [19] to treat balance and PC disorders in patients with MS. This gaming protocol proposes activities that involve integrating proprioceptive, visual, and vestibular sensory information. Responses directed to the maintenance of balance and postural stability are triggered by the visual feedback that patients continuously receive in real time with regard to their position, performance type, and the movement direction that the task requires.

The protocol proposed tasks such as throwing and hitting objects with one’s hands and feet, hitting and receiving balls with different body parts, dodging objects, overcoming obstacles, imitating postures, or managing virtual elements that favor key aspects of PC (e.g., girdle dissociation, alternating load distribution, changes in direction, multidirectional movement, reaction speed, hand-eye coordination, foot-eye coordination, and dexterity) in different positions across a stepwise gradient of difficulty. The software raises the gaming difficulty level depending on patient results and progress.

Participants attended 40 sessions at intervals of four sessions per week (20 min per session). There was a progressive increment based on individual patients’ fatigue level, up to 20 min per session. The home television sets of patients were used as the interface for the video games. Patients were advised to conduct the gaming sessions when another person was at home to minimize the potential risks associated with the treatment protocol. A physiotherapist monitored and supervised all interventions on real time using online meetings via videoconferencing to avoid adverse events. The treatment schedule lasted 10 weeks for both groups.

Computerized dynamic posturography (CDP) was used to evaluate all patients at baseline and at the end of the treatment protocol. The CDPs were conducted at the Motion Analysis, Biomechanics, Ergonomics, and Control Laboratory (LAMBECOM) at Rey Juan Carlos University. This study employed the Smart EquitestTM Version 8.2 CDP device (NeuroCom International Inc., Clackamas, OR, USA). CDP is a quantitative method used to evaluate and treat balance disorders [20,21]. This device consists of: (1) two force plates with pressure-sensitive strain gauges located in each quadrant that translate horizontal, vertical, and rotational movement using an axis collinear with the ankle joint; (2) a movable visual surface that encloses patients and rotates in a forward-backward direction parallel to the floor; and (3) a computer that processes data. The dynamometric platform measures pressure-center displacement using sensors that register the different pressures exerted by the body in static and dynamic situations. CDP protocols assess sensory and motor (automatic and voluntary) impairments. 

The Sensory Organization Test (SOT) is a specific CDP test considered by several authors as the “gold standard” for studying PC [22]. Furman [23] indicated that SOT has a sensitivity of 95% with a range of 5% false positives, and Di Fabio [24] identified a specificity of 92% for the sensory organization component of CDP. The SOT provides an extremely sensitive assessment of the major sensory systems involved in balance and stability [25]. The SOT assesses balance and PC by stimulating the visual and proprioceptive systems. These sensory systems are used in a combined and variable manner to calculate the degree of functional impairment and the compensation of the different systems involved in balance control. Individuals stand on a force platform surrounded by a visual enclosure. Both the platform and visual display can be stationary or sway-referenced to the individual’s bodily movement. In the latter condition, the center of gravity (COG) shifts in the anterior-posterior direction are accompanied by a forward or backward rotation of the force plate, the visual display, or both. The sway referencing of the platform renders the somatosensory information recorded from the ankle joints inaccurate, which causes the participant to rely more heavily on visual, vestibular, or both types of inputs. Likewise, the sway referencing of the visual display renders the visual information (which is relevant for balance control) inaccurate, which causes the participant to rely more heavily on somatosensory, vestibular, or both types of inputs. In the SOT, participants must maintain a stable COG in three consecutive 20-s series for all six conditions in the test. In the first three conditions, the platform remained fixed. Individuals participated with their eyes open, closed, and with a mobile visual environment referenced to postural oscillations in Conditions 1 (SOT1), 2 (SOT2), and 3 (SOT3), respectively. Conditions 4 (SOT4), 5 (SOT5), and 6 (SOT6) repeated the visual conditions of the first three tests and added a platform movement referenced to the anteroposterior oscillation of the participant with the ankle-foot angle remaining constant, thereby negating proprioceptive sensory input [26]. 

Participant height in centimeters (without shoes) and body mass in kilograms were measured. Participants stepped onto the platform base with assistance, where they faced the visual display. They wore comfortable, loose clothing and socks. Each foot was positioned on one force plate such that the medial malleolus and lateral portion of the calcaneus were aligned with the appropriate markers according to the CDP instructions. Participants stood upright with their arms at their side to face the visual display and maintained their balance throughout the SOT. Each participant was fit with a harness that was secured to an overhead bar with straps prior to testing. The harness was fit in such a way that it would support participants if they fell but would not support them in a normal, upright stance or during balance adjustments.

The dependent variables for the SOT included the composite equilibrium score (CES), sensory analysis, and sensory ratios. CES quantified the COG sway or postural stability in each of the six sensory conditions across three trials. The effective use of individual sensory inputs was determined from the overall pattern of scores across the six conditions. The CES and the weighted average of all 6 individual scores (with the first two conditions carrying a weight of 1/14 and the other four conditions carrying a weight of 3/14 [26]) characterized overall level of performance. Values close to 100% indicate controlled balancing, and those close to 0% indicate a fall. 

The sensory analysis depicts the sensory ratios computed from the average equilibrium scores obtained from specific sensory test condition pairs [27,28]. Somatosensory ratio (SR; (SOT2/SOT1) × 100) was used to assess participant ability to use the input from the somatosensory system to control balance. Visual ratio (VR; (SOT4/SOT1) × 100) assessed patient ability to use the input from the visual system to maintain balance. The vestibular ratio (VEST; (SOT5/SOT1) × 100) assessed the ability to use the input from the vestibular system to maintain balance, and the visual preference ratio (PREF; (SOT 3 + 6/SOT 2 + 5) × 100) assessed the degree to which patient relies on visual information to maintain balance, even when the information is incorrect [27,28].

All participants in this research were informed of the protocol’s objectives and risks. Patients who met the inclusion criteria volunteered for this research by providing written consent. The Research Ethics Committee of the San Carlos University Hospital, Madrid, approved this study in March 2011.

The data analyses were performed using the software package SPSS Version 19.0. The first analysis addressed the features and regularities of the dataset using descriptive statistics of the qualitative variables (*i.e*., frequency distributions) and the quantitative variables (*i.e*., means and standard deviations; SDs). The Kolmogorov-Smirnov test was used to determine whether the study variables were normally distributed (*p* > 0.05). An independent-samples *t*-test was performed for each quantitative variable to determine the homogeneity of the sample. A Levene’s test *p*-value less than 0.05 was considered significant. A paired-samples *t*-test was used to analyze the pre- and post-intervention differences in the balance measurement variables (SOT, and sensory analysis) within groups. An analysis of variance (ANOVA) was used to compare the pre- and post-intervention differences using the group parameters (EG and CG) as the between-subject factor and the study variables as within-subject factors. Variables that did not meet the homogeneity criteria (according to the visual information) were analyzed using an ANOVA with the baseline variable as an additional covariate. The significance threshold was set at *p* ≤ 0.05, and 95% confidence intervals were obtained for each test.

## 3. Results

Forty-seven patients (27 women and 20 men) between the ages of 28 and 60 years completed this research study. Two participants in the CG dropped out, and one in the EG dropped out due to an outbreak. The mean age of the CG (*n =* 23) was 42.78 ± 7.38 years (mean ± SD), with 10.86 ± 5.40 years since MS diagnosis. The mean age of the EG (*n =* 24) was 39.69 ± 8.13 years, with 9.68 ± 6.76 years since MS diagnosis. Table 2 displays the other sociodemographic variables, the data related to progress, and the expanded disability status scale (EDSS) scores. The groups did not differ significantly at baseline with respect to age, duration of disease, or type of MS (Table 2). 

**Table 2:** Sociodemographic characteristics.

Variable	Group	Mean	SD	*p*-value
Age (years)	Experimental Control	39.69	8.13	
		42.78	7.38	0.061
Sex	Experimental Control	Women 54.2 % (*n =* 13)		
		Men 45.8% (*n =* 11)		
		Women 60.9% (*n =* 14)		
		Men 9.1 % (*n =* 9)		
Years since diagnosis	Experimental Control	9.68	6.76	
		10.86	5.40	0.142
MS type	Experimental Control	PP 20.0 % (*n =* 5)		
		RR 71.9 % (*n =* 16)		
		SP 8.1 % (*n =* 3)		
		PP 8.7% (*n =* 2)		
		RR 65.2% (*n =* 15)		
		SP 26.1% (*n =* 6)		
EDSS	Experimental Control	Score 3 EDSS 16.4 % (*n =* 4)		
		Score 4 EDSS 75.5 % (*n =* 17)		
		Score 5 EDSS 8.1 % (*n =* 3)		
		Score 3 EDSS 21.7 % (*n =* 5)		
		Score 4 EDSS 60.9 % (*n =* 14)		
		Score 5 EDSS 17.4 % (*n =* 4)		

MS: Multiple Sclerosis; PP: Primary Progressive; RR: Relapsing Remitting; SP: Secondary Progressive.

The contrast statistical analysis of the baseline results did not reveal significant between-group differences with regard to the balance variables. Thus, we considered the groups to be homogeneous with regard to the comparative analyses discussed below. 

### 3.1. Pre- and Post-Intervention within-Group Comparisons

A paired-samples *t*-test was used to analyze the CES percentages of the EG from pre- to post-intervention. The results indicated significant differences, with an increase of 8.21 points from the pre-treatment baseline to the post-treatment evaluation (*p* < 0.001). Conversely, this test was not significant for the CG (*p* = 0.123; Table 3).

**Table 3:** *t*-test results of the within-group differences from pre- to post-treatment.

Variable	Group	PRE-TREATMENT			POST-TREATMENT			F	Sig.
		Mean ± DE	95% IC		Mean ± DE	95% IC			
			Min.	Max.		Min.	Max.		
**CES**	Control	62.85 ± 12.17	58.48	67.23	64.78 ± 9.70	61.34	69.22	37.873	**<0.001**
	Experimental	62.37 ± 11.35	58.02	66.72	70.58 ± 9.68	66.07	75.09		
**ViR**	Control	85.58 ± 8.68	79.81	91.36	85.92 ± 7.36	81.39	90.45	2.909	0.095
	Experimental	85.27 ± 17.26	79.62	90.92	87.57 ± 8.44	82.89	92.24		
**PREF**	Control	121.55 ± 23.93	110.55	132.55	120.55 ± 25.37	111.18	129.93	15.051	**<0.001**
	Experimental	117.82 ± 23.68	108.03	127.61	133.11 ± 20.79	122.96	143.27		
**VEST**	Control	45.44 ± 12.45	42.48	48.41	45.02 ± 17.79	41.43	48.61	12.156	**<0.001**
	Experimental	40.54 ± 19.93	33.68	47.41	53.28 ± 15.85	46.36	60.20		
**SR**	Control	89.29 ± 11.08	85.04	93.54	93.76 ± 7.20	89.99	97.50	0.117	0.734
	Experimental	90.70 ± 9.12	86.20	93.98	92.47 ± 8.67	89.42	95.53		

Significant (*p* < 0.05); CES: Composite Equilibrium Score; PREF: Visual Preference Ratio; VEST: Vestibular Ratio; ViR: Visual Ratio; SR: Somatosensory Ratio.

The sensorial analysis of the SOT results provided information on the participation and use of each sensory system with regard to maintaining proper balance. Furthermore, the results of the *t*-tests of visual preference and the contribution of vestibular information yielded significant differences (*p* < 0.001) in the EG. Conversely, significant differences were not found with regard to the contribution of visual and somatosensory information (*p* > 0.05). Furthermore, only the contribution of the somatosensory input source significantly differed in the CG (*p*= 0.043).

### 3.2. ANOVAs

An ANOVA revealed significant, between-group post-treatment differences in the CES percentage from the SOT (F = 37.873, *p* < 0.001). Moreover, an ANOVA yielded significant between-intervention-group differences in the contribution of the vestibular system (F = 12.156, *p* < 0.001), which demonstrates that the EG made better use of this information at post-intervention compared with the CG. In addition, significant differences were found with regard to the ability to accept incorrect visual information expressed by the visual conflict parameter (F = 15.05, *p* < 0.000), which demonstrates that the EG showed a greater ability to accept these post-treatment afferent inputs compared with the CG. Furthermore, an ANOVA did not reveal significant between-group differences with regard to the contribution of the visual system (F = 2.64, *p* = 0.11) or use of somatosensory information (F = 0.117, *p* = 0.734) in the maintenance of balance and stability. The statistical accuracy of the five variables included in the SOT is shown in Table 4 with 95% confidence intervals.

**Table 4:** Descriptive statistics and marginal measures for pre- and post-treatment ANOVA between groups.

Variable	Group	PRE-TREATMENT			POST-TREATMENT			F	*p*
		Mean ± SD	95% CIs		Mean ± SD	95% CIs			
			Min.	Max.		Min.	Max.		
**CES**	CG	62.85 ± 12.17	58.48	67.22	64.78 ± 9.70	61.33	69.22	37.873	0.000 *
	EG	62.37 ± 11.35	58.02	66.72	70.58 ± 9.68	66.07	75.09		
**ViR**	CG	85.58 ± 8.68	79.81	91.36	85.92 ± 7.36	81.39	90.46	2.909	0.095
	EG	85.27 ± 17.26	79.61	90.92	87.57 ± 8.44	82.89	92.24		
**PREF**	CG	121.55 ± 23.93	110.55	132.55	120.55 ± 25.37	111.18	129.93	15.051	0.000 *
	EG	117.82 ± 23.68	108.03	127.60	133.13 ± 20.79	122.96	143.27		
**VEST**	CG	45.44 ± 12.45	42.47	48.41	45.02 ± 17.79	41.43	48.616	12.156	0.000 *
	EG	40.54 ± 19.93	33.68	47.41	53.28 ± 15.85	46.37	60.203		
**SR**	CG	89.29 ± 11.08	85.03	93.54	93.76 ± 7.20	89.99	97.501	0.117	0.734
	EG	90.37 ± 9.12	86.20	93.98	92.48 ± 8.67	89.42	95.534		

* Significant (*p* < 0.05; expressed as mean difference; 95% CIs. min-max); CES: Composite Equilibrium Score; PREF: Visual Preference Ratio; VEST: Vestibular Ratio; ViR: Visual Ratio; SR: Somatosensory Ratio.

## 4. Discussion

To our knowledge, this article is the first to evaluate the implementation of a TR program using a virtual in-home therapy to improve balance and PC in patients with MS. Previous authors have studied the effect of semi-immersive VR video game systems on PC and balance disorders in adult patients with various neurological conditions [14,29,30,31]. However, only one study evaluated the balance and PC of patients with MS using VR systems [29]. Importantly, all of the studies cited above were conducted in the context of outpatient rehabilitation, except for Cikajlo *et al*. [31], who conducted their study with patients with CVA. 

Results showed an improvement over general balance in both groups. Visual preference and the contribution of vestibular information only yielded significant differences in the experimental group. The results of this study demonstrated improvements in the balance and PC of patients with MS after completing either a TR program using video game VR technology or a conventional rehabilitation program. With regard to the specific study objectives, a TR program might be an important alternative to standard rehabilitation treatments for the balance and PC of patients with MS who have problems with mobility, geographical access, or both [8]. Our study revealed significant improvements in the EG related to overall balance, vestibular and visual preference sensory inputs, and improved automatic postural response.

No participants achieved a CES above 70% at baseline. This cut-off score is the normal minimum value (*i.e*., the value achieved by 95% of the age-matched population without symptoms or a history of disequilibrium) [32]. After the intervention, only participants in the EG reached CESs above 70%.

Initially, the evaluation of the CESs from the SOT showed significant within-group and between-group improvements in the EG and not the CG. Importantly, Peterson *et al*. [33] described a possible learning effect and found a 10% increase in the equilibrium capacity related to repeated exposure to the SOT. Likewise, Guskiewicz *et al*. [34] specified that a change of 6.83 in the CES over baseline is needed. Fatigue is an important symptom of MS and may mask the results, therefore, we decided not to repeat the posturography tests. However, the mean values in our study exceeded that cut-off; thus, we concluded that the improvements occurred due to the experimental intervention (an 8.20-point increase occurred from the baseline in the EG.)

Improved balance and PC in the EG might be related to the motor training principles addressed by Shumway-Cook [35], which are listed: (a) Increasing the level of practice in a distributed manner; (b) Increasing functional task repetition; (c) Sensory feedback modality used. With respect to the use of vestibular information, however, the results of this research showed that virtual training improves the ability to use these inputs to maintain balance and PC. These results were not observed in the CG. Pavlou *et al*. [36] showed how the visual-vestibular conflict (which is characteristic of virtual environments) considerably compromises the maintenance of balance and PC and increases the use of vestibular information integration in healthy participants. This finding might be related to the angular displacement of the head when trying to adapt one’s gaze to bodily movement trajectories and interact with objects in a virtual environment.

In addition, a better visual preference parameter response was observed in the EG compared with the CG. Patients with MS lose stability while viewing erroneous optokinetic stimuli in an open and changing visual environment. As a result, VR training offers the possibility of integrating multiple visual inputs across different visual field levels with stimuli that have high variability in their direction, path, and speed, thereby significantly increasing the number of reliable peripheral visual stimuli. Yen *et al*. [30] also came to this conclusion in their research with patients with Parkinson’s disease. 

Moreover, balance and PC involve not only maintaining stability but also recovering from disequilibrium. Thus, the activation of the automatic response mechanisms that ensure the recovery of stability is needed to prevent falling. Authors such as Kuno *et al*. [37] have reported that balancing the body in virtual environments is proportional to the velocity of the perceived visual stimulus, and COG stabilization depends on the fast and accurate integration of visual inputs. Thus, contrary to conventional therapy in which the environment remains constant or is modified with minor variations, virtual environments are dynamically configured and variable [38,39]. Therefore, the visual demands of the environment require one to constantly readjust the perceived spatial and temporal information (*i.e*., to predict the body’s position in space and the direction of motion that directly activates feed-forward postural mechanisms). The constant readjustment of visual information required for recovering stability also necessitates constant feedback. In this respect, video game and VR systems inform the participants about their movements and their degree of accuracy, and the results of their actions on the environment allow them to train their adaptive reflex responses to ensure the restoration of balance and PC [40]. 

This research has several limitations. First, participants were selected using non-probabilistic sampling; a discretionary sample was used with pre-set criteria including area of residence, access to rehabilitation services, and whether rehabilitation treatment was available. Second, the research was not blind; however, an independent evaluator assessed balance and PC. Third, no follow-up was performed to conclude that the improvements remained stable over time. Finally, the VR system proposed activities were not specifically designed to serve a rehabilitative purpose. However, experts in treating MS developed the exercise protocol according to criteria of playability with the aim of adjusting the protocol to the balance and PC of patients with MS.

## 5. Conclusions

Our results demonstrated that a TR program based on a VR system allows one to optimize the sensory information processing and integration systems necessary to maintain the balance and PC of people with MS. In addition, we suggest that the VR program discussed in this research enables anticipatory PC and response mechanisms (in the event of a stability disturbance) and might serve as a successful therapeutic alternative in situations in which conventional therapy is not readily available. Additional research is needed to: (a) evaluate the ability of these systems to treat other symptoms associated with MS (e.g., coordination, muscle strength, and manipulative skills); (b) validate the use of online platforms and adapted video game systems with regard to the neurological condition rehabilitation therapies and develop TR assistive devices to suit the needs of patients with MS; (c) ensure that the design of these platforms includes a task repertoire that is sufficiently broad to develop comprehensive and personalised treatment programs; and (d) evaluate the real effect of these programs by analysing their cost-effectiveness to enable their delivery at a sustainable cost.
